# Pam3CSK4 As a Second Hit in NLRP3‐Dependent Activation of Monocytes Primed With Antiphospholipid Antibodies

**DOI:** 10.1002/eji.70245

**Published:** 2026-07-22

**Authors:** Anush Martirosyan, Eva Kriegova, Jana Ulehlova, Zaven Karalyan, Tomas Papajik, Gayane Manukyan

**Affiliations:** ^1^ Laboratory of Molecular and Cellular Immunology Institute of Molecular Biology National Academy of Sciences Yerevan Armenia; ^2^ Department of Immunology Faculty of Medicine and Dentistry Palacký University Olomouc and University Hospital Olomouc Olomouc Czech Republic; ^3^ Department of Hematology‐Oncology Faculty of Medicine and Dentistry Palacký University Olomouc and University Hospital Olomouc Olomouc Czech Republic; ^4^ Laboratory of Cell Biology and Virology Institute of Molecular Biology National Academy of Sciences Yerevan Armenia

**Keywords:** antiphospholipid antibodies, IL‐1β, NLRP3 inflammasome, Pam3CSK4, tissue factor, TLR1/2, TLR4, THP‐1 cells

## Abstract

Antiphospholipid antibodies (aPL) are drivers of inflammation and thrombosis in antiphospholipid syndrome (APS). However, the molecular mechanisms underlying infection‐induced exacerbation of APS remain incompletely understood. To identify novel pathways contributing to the pro‐inflammatory and pro‐coagulant activation of monocytes in APS, we screened fourteen Toll‐like receptor (TLR) ligands for their ability to induce tissue factor (TF) expression in a monocyte‐like cell line (THP‐1). Among these, Pam3CSK4, a synthetic TLR1/2 ligand, emerged as a potent inducer of TF in the presence of aPL. Subsequent experiments revealed that Pam3CSK4 synergized with aPL to amplify both TF expression and IL‐1β secretion. This synergistic effect was mediated via NF‐κB signaling and required activation of the NLRP3 inflammasome. aPL priming promoted ATP release, providing the second signal for NLRP3 activation. Inhibition of NLRP3 markedly reduced Pam3CSK4‐induced TF expression, suggesting a critical role for inflammasome signaling in monocyte procoagulant responses. These in vitro findings were confirmed in a mouse model of APS, supporting the pathogenic relevance of the TLR1/2–NLRP3 axis in vivo. Taken together, our results validate the “second hit” paradigm in APS and suggest a novel TLR1/2–NLRP3 axis as a key mediator of infection‐driven inflammatory and thrombotic responses in aPL‐primed monocytes.

## Graphical abstract

Pam3CSK4 amplifies aPL‐induced monocyte activation. This results in thromboinflammatory activation that may contribute to APS‐related vascular pathology.

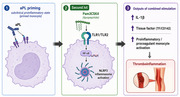

## 1 Introduction

Antiphospholipid syndrome (APS) is a multisystemic autoimmune disorder that arises in the setting of persistent antiphospholipid antibodies (aPL). Clinical manifestations include arterial or venous thrombosis and/or recurrent early pregnancy loss or other pregnancy‐related complications [1, 2, 3]. The main autoantigen in APS is β2‐glycoprotein I (β2GPI), also known as apolipoprotein H, with anti‐β2GPI antibodies recognized as central pathogenic drivers [4]. Anti‐β2GPI antibodies are thought to be responsible for the alterations in hemostasis and coagulation via interference with the anticoagulatory components [5], activation of platelets [6], monocytes [7], and endothelial cells [8], eventually leading to a proinflammatory or hypercoagulable state. Although anti‐β2GPI is widely considered a key trigger of APS manifestations, its mere presence has shown a weak independent correlation with thrombosis and inconsistent associations with obstetric complications [9]. The traditional explanation for this phenomenon is the “two‐hit theory”, which proposes that the aPL‐mediated procoagulant state constitutes the “first hit,” while an additional triggering factor, the “second hit”, is required to elicit clinical manifestations of APS [10]. Infections are considered among the most significant environmental factors responsible for both the induction of aPL and the transition to clinically manifest APS [11]. In patients with defined APS, concomitant activation of innate immunity, particularly via pathogen‐sensing Toll‐like receptors (TLRs), has been shown to promote thrombus formation [11, 12].

In recent years, numerous studies have focused on the pathological mechanism underlying the TLR4/β2GPI interplay in the context of APS, yielding significant insights. It has been observed that anti‐β2GPI/β2GPI complexes elicit a signaling cascade that converges with canonical LPS‐induced responses, most notably the TLR‐4/MyD88/ NF‐κB axis [12, 13, 14, 15]. This phenomenon may be driven by structural similarities between human β2GPI and bacterial lipopolysaccharide (LPS) [16], or by the capacity of cell‐bound β2GPI to promote the assembly of receptor‐associated signaling complexes at the plasma membrane [13, 17, 18]. These observations provided an assumption that TLR4 might function as an “adaptor” for intracellular signal transduction by anti‐β2GPI. In monocytes [13] and trophoblasts [19], anti‐β2GPI antibodies trigger NF‐κB‐dependent gene transcription by inducing the co‐localization of TLR4 and surface‐bound β2GPI, leading to a proinflammatory phenotype relevant to APS pathogenesis. In endothelial cells, which do not naturally express β2GPI, circulating β2GPI can bind to cell surface receptors such as Annexin A2 and ApoER2, facilitating the assembly of signaling complexes that include TLR4 and associated molecules, ultimately leading to endothelial cell activation [14, 18]. This model is further supported by the biophysical properties of β2GPI itself: while circulating β2GPI binds negatively charged phospholipids weakly, anti‐β2GPI antibody‐induced dimerization markedly enhances its affinity for phospholipid surfaces, thereby favoring stable membrane engagement and more efficient signal initiation [20].

To date, all related studies have focused solely on the TLR4 engagement in a pathological context of aPL‐positivity. However, infectious triggers implicated in APS are diverse [11], and it remains unclear whether innate immune pathways beyond TLR4 can similarly cooperate with aPL to promote pathogenic cellular activation. Among pattern‐recognition receptors, TLRs are particularly relevant because they are expressed by APS‐associated target cells, including monocytes, endothelial cells, and trophoblasts, and their capacity to directly sense diverse microbial products associated with infection‐related “second hits” [13, 14, 19, 21, 22].

We therefore hypothesized that innate immune stimuli beyond the classical TLR4 agonists may also contribute to the procoagulant and inflammatory state observed in APS. To address this knowledge gap, we systematically assessed a broad panel of well‐known TLR ligands and selected innate immune stimuli for their ability to modulate aPL‐mediated activation of monocytes, a key effector cell type in APS. The current study identified a novel pathogenic TLR ligand capable of synergizing with anti‐β2GPI antibodies to amplify pro‐coagulatory and pro‐inflammatory responses. We further investigated the underlying molecular mechanisms driving these interactions and complemented the in vitro experiments with a mouse model of APS to determine whether these effects also translate into enhanced pathogenic responses in vivo.

## 2 Materials and Methods

### 2.1 APL IgG Isolation

Anti‐β2GPI‐positive and anti‐β2GPI‐negative IgG were isolated from mice with an APS model and healthy control animals, respectively. The mice were housed in standard cages (1/cage) in a controlled environment, with a relative humidity of 60 ± 5%, a 12 h/12 h light–dark cycle, and a constant temperature of 25 ± 2°C. Animals had ad libitum access to tap water and rodent chow throughout the whole study. All methods are reported in accordance with ARRIVE guidelines. Euthanasia methods comply with AVMA standards. Mice were kept under conditions according to protocol #05072021/1; 07/05/2021, issued by the Committee of the Institute of Molecular Biology of the National Academy of Sciences of the Republic of Armenia for the Care and Maintenance of Animals. All methods were carried out in accordance with relevant guidelines and regulations.

To establish the APS animal model, we adopted the β2‐glycoprotein I (β2GPI) immunization protocol, which has been demonstrated to induce high anti‐β2GPI titer and APS‐like manifestations in mice, as reported in our recent study [23]. Briefly, the APS animal model was generated in BALB/c mice via a three‐step immunization 10 days apart. Mice (12 weeks old, weighing 20 ± 2 g) were immunized with solubilized Human Apolipoprotein H Native Protein (β2GPI, Invitrogen) (10 µg/per injection) emulsified in equal amounts with complete Freund's adjuvant (Sigma). Mice from the control group were receiving injections with an adjuvant. Anti‐β2GPI positive status was confirmed with in‐house ELISA. For this, a high absorbance 96‐well plate with a hydrophilic/hydrophobic mix surface was coated overnight with 3 µg/mL β2GPI, washed, and blocked with 1% BSA/PBS solution for 1h and incubated with 50 µL of 1:500 diluted mouse sera for 1 h. Next, the plate was incubated with streptavidin conjugated goat anti‐mouse secondary IgG (R&D Systems). Colorimetric reaction was achieved by the addition of TMB substrate (R&D Systems). Following 15 min incubation, the reaction was stopped, and absorbance was measured with an ELISA plate reader at 450 nm. Determination of antibody levels was performed with freshly obtained sera samples. Following the confirmation of anti‐β2GPI‐positivity, animals were selected for plasma collection to isolate total anti‐β2GPI containing IgG using the Protein G SpinTrap (Cytiva) according to the manufacturer's instructions. IgG concentration was measured using a NanoQ (Mecasys). All animals selected as anti‐β2GPI‐negative IgG donors were also tested for anti‐β2GPI status.

### 2.2 Cell Culture

Experiments were performed with the use of THP‐1 cells, a human myelomonocytic cell line obtained from the American Type Culture Collection (ATCC TIB‐202). The cells were maintained in endotoxin‐free RPMI medium supplemented with 10% FBS, 2 mM L‐glutamine, and a mixture of antibiotics (5 mg/mL penicillin and 5 mg/mL streptomycin). Cells were cultured at 37°C in a humidified atmosphere containing 5% CO^2^.

### 2.3 Solid Phase Assay

Cell activation with anti‐β2GPI IgG complexed with β2GPI was performed using a solid‐phase assay. Briefly, a high absorbance 96‐well plate (Nunc MaxiSorp ELISA Plates, Uncoated, Biolegend) with a hydrophilic/hydrophobic mix surface was coated overnight with 2 µg/mL recombinant human β2GPI. The next day, wells were washed with PBS, blocked with 1% BSA/PBS solution for 1 h, and then incubated with either anti‐β2GPI‐positive IgG (50 µg/mL), anti‐β2GPI‐negative IgG (50 µg/mL) antibodies, or vehicle control for 1 h. For the experiments, THP‐1 cells (50,000/well) were seeded on freshly prepared plates with immobilized anti‐β2GPI‐positive IgG/β2GPI or control anti‐β2GPI‐negative IgG/β2GPI complexes in the absence or presence of indicated stimulators and incubated for a total of 4 h at 37°C in a humidified atmosphere containing 5% CO_2_. This scheme was utilized for all the experiments described below, if not otherwise mentioned. Complex formed with aPL+ IgG/β2GPI will be hereafter referred to as aPL+, and complex of aPL− IgG/β2GPI as aPL‐.

### 2.4 TLR‐Ligand Screening

Before the selection of Pam3CSK4 as ligand of interest, an evaluation of a range of TLR‐ligands and other infection‐related agents was conducted to assess their ability to potentiate aPL+ induced tissue factor (TF, surface marker CD142) expression. For this, THP‐1 cells were exposed to a panel of following agents: 1 µg/mL peptidoglycan (PGN, Sigma), 10 µg/mL Concanavalin A (ConA, Invivogen), 10 µg/mL polyinosinic:polycytidylic acid (Poly I:C, Sigma), 2.5 µg/mL resiquimod (R848, Invivogen), 100 ng/mL fibroblast‐stimulating lipopeptide (FSL1, Sigma), 100 ng/mL lipopolysaccharide (LPS, Sigma), 5 µg/mL lipoteichoic acid (LTA, Invivogen), 200 ng/mL tripalmitoylated lipopeptide (Pam3CSK4, Invivogen), 10 ng/mL CpG DNA (Invivogen), 100 ng/mL lipoarabinomannan (LAM, Invivogen), 10 µg/mL muramyl dipeptide (MDP, Invivogen), 5 µg/mL single‐stranded RNA40 (ssRNA40, Invivogen), 10^7^cell/mL heat‐killed preparation of Listeria monocytogenes (HKML, Invivogen), and 100 nM N‐Formyl‐Met‐Leu‐Phe (fMLP, Sigma) on the plate with immobilized anti‐β2GPI/β2GPI. After a 4 h cultivation period, the cells were collected, stained with CD142, and analyzed with flow cytometry.

### 2.5 Pam3CSK4 Stimulation

To further investigate the mechanism underlying Pam3CSK4‐mediated enhancement of aPL+ induced hypercoagulability, cells were stimulated with aPL+ or aPL− in the absence or presence of Pam3CSK4 (200 ng/mL) for 4 h. Following this incubation period, surface expression of TF (CD142), CD54, TLR1 (CD281), TLR2 (CD282), TLR4 (CD284), and CD14 was evaluated by flow cytometry using a NovoCyte (Agilent).

As a positive control for NLRP3 inflammasome activation, cells were subjected to a two‐step stimulation with LPS (100 ng/mL) for 3 h, followed by 5 µM Nigericin (Invivogen) for a further 1 h. Surface expression of TF (CD142) was measured with flow cytometry and IL‐1β release by ELISA. As a negative control, cells were treated with CP‐456,773 (50 µM). CP‐456,773 (also known as MCC950) is a selective NLRP3 inhibitor that binds to its NACHT domain, blocking ATP hydrolysis and oligomerization. This prevents inflammasome assembly and suppresses IL‐1β maturation and release.

### 2.6 TLR Blocking Assays

To investigate the contribution of TLR2 and TLR4 in the observed effects, THP‐1 cells were pretreated for 30 min with TL2‐C29 (100 µM; TLR2/1 and TLR2/6 inhibitor, Invivogen), CLI‐095 (1 µM; TLR4 inhibitor, Invivogen), or their combination. Following pretreatment with TLR blocking agents, cells were subjected to the solid‐phase stimulation under the same conditions as noninhibited cells, using Pam3CSK4 (200 ng/mL) either alone or in combination with aPL− or aPL+. After cultivation, cells were immediately analyzed with flow cytometry for surface TF expression, and supernatants were collected and stored for IL‐1β quantification.

### 2.7 Flow Cytometry

Surface expression of TF(CD142)‐PE, TLR2 (CD282)‐PeCy7, TLR4 (CD284)‐APC, CD14‐PerCP, CD54‐FITC (all from Biolegend), and TLR1 (CD281)‐BV421 (BD Biosciences) was assessed by flow cytometry using NovoCyte flow (Agilent) and LSR II flow cytometers (BD Biosciences). After stimulation, cells were harvested and stained with fluorochrome‐conjugated antibodies according to standard procedures, washed, and acquired on the instrument. Data were analyzed using FlowJoTM (version 10.1; BD Biosciences) software, and results were expressed as the percentage of positive cells and/or MFI.

### 2.8 Fluorescent Staining

THP‐1 cells stimulated with aPL− or aPL+ in the absence or presence of Pam3CSK4 were stained for NLRP3 as follows. 96‐well plates with prestimulated cells were centrifuged, and culture supernatants were carefully removed. Cells were fixed with 4% (w/v) formaldehyde solution for 20 min, washed, and permeabilized with 0.5% Triton X‐100 (v/v) in PBS. After additional washing steps, blocking was performed overnight at 4°C using 5% BSA in PBS. The next day, cells were washed and incubated with 5 µg/mL rat anti‐human NLRP3 antibodies (ThermoFisher) for 1h, followed by 1h incubation with goat anti‐rat secondary antibodies, conjugated with Alexa 488 (Abcam). After final washes, cells were counterstained with DAPI, and images were taken with BioTek Cytation C10 Confocal Imaging Reader.

### 2.9 Caspase‐1 Activity Assay

Active caspase‐1 was assessed using a FLICA 660 CASPASE‐1 KIT (BioRad), according to the manufacturer's instructions. FLICA 660 is a cell‐permeant caspase‐1 probe based on the YVAD recognition sequence, conjugated to a far‐red 660 dye and an FMK reactive group that covalently binds active caspase‐1. THP‐1 cells stimulated as described above were used for caspase‐1 activity assessment. As a positive control, THP‐1 cells were treated with LPS (100 ng/mL) for 3 h, followed by stimulation with Nigericin (5 µM) for an additional 1 h. Cells were incubated with the FLICA 660 working solution for 60 min at 37°C in the dark. Stained cells were analyzed on a NovoCyte flow cytometer.

### 2.10 ATP Measurements

Measurement of ATP release was performed in THP‐1 seeded in aPL− or aPL+ coated wells in the absence or presence of Pam3CSK4 (200 ng/mL). Components of the Luminescent ATP Detection Assay Kit (AbCam) were added to cells, according to the manufacturer's instructions. Luminescence was recorded during the first 60 min of stimulation using the BioTek Cytation C10 Confocal Imaging Reader to assess dynamic ATP levels.

### 2.11 LDH Assay

The LDH assay, a method for determining cytotoxicity, measures the activity of the cytoplasmic enzyme lactate dehydrogenase (LDH) released from damaged cells. LDH levels in the cell culture supernatants were quantified using the CytoTox 96 Non‐Radioactive Cytotoxicity Assay (Promega), according to the manufacturer's instructions.

### 2.12 Adhesion Assay

The expression of TF by activated monocytes is one of the key events that triggers the initiation of the coagulation cascade. TF may facilitate the adhesion of monocytes to endothelial cells, which further enhances their procoagulant activity. To assess the impact of aPL+ and Pam3CSK4 on THP‐1 ability to adhere, we performed a dynamic adhesion assay. For this, HUVEC/TERT 2 cells (ATCC) were grown to 80% confluence on 48‐well plates using Vascular Cell Basal Medium supplemented with Endothelial Cell Growth Kit‐VEGF (ATCC). Immediately before assay, the HUVEC monolayer was depleted from the growing medium and washed. Prestimulated THP‐1 cells were labeled with CFSE, resuspended at a final concentration of 1 × 10^5^/100 µL PBS, and loaded on HUVEC. The plate was placed on a horizontal shaker operating at 210 strokes/min, and cells were allowed to adhere for 15 min. Unattached cells were removed with double PBS rinsing. Adherent cells were imaged using a microscope imaging system (Agilent BioTek Cytation C10) in the montage mode (16 locations). Counting was performed automatically with the use of BioTek Gen5 3.14 Software.

### 2.13 ASC‐Speck Formation Assay

ASC‐speck formation was analyzed by flow cytometry using intracellular ASC staining. After stimulation under the indicated experimental conditions, cells were harvested, washed with PBS, fixed with 4% (w/v) formaldehyde solution for 30 min, followed by permeabilization with 0.5% Triton X‐100 (v/v) in PBS for 30 min. Cells were then stained with an anti‐ASC‐PE antibody (BioLegend) for 1 h, followed by washing and acquisition by the LSR II flow cytometer.

### 2.14 Calcium Flux Assay

The intracellular Ca^2^^+^ content and inducible flux were measured as the fluorescence intensity of Fluo‐4 AM (Invitrogen). At the end of a 4 h treatment with aPL−/aPL+ and Pam3CSK4 in a solid‐phase assay, THP‐1 cells were washed, resuspended in PBS containing 2 µM Fluo‐4 AM and 0.02% Pluronic F‐127, and incubated for 30 min at 37 °C in the dark. Following incubation, the cells were washed, resuspended in Ca^2^^+^/Mg^2^^+^‐containing PBS, and incubated for an additional 30 min at 37 °C in the dark before measurement. During the measurement, the basal level of intracellular Ca^2^^+^ was assessed for 30 s, after which a Ca^2^^+^ ionophore (Ionomycin, 1 µg/mL) was added to the sample, and fluorescence was recorded immediately for an additional 3 min. Green fluorescence was analyzed using an LSR II flow cytometer (BD Biosciences).

### 2.15 ELISA

Levels of soluble IL‐1β in cell culture supernatants were measured using the ELISA MAX Deluxe Set for Human IL‐1β (BioLegend), according to the manufacturer's instructions.

### 2.16 Animal Model for Aps and Pam3sck4 Administration

For this part of the study, twelve 12‐week‐old female BALB/c mice were used. Animals were divided into three groups: four mice as the healthy control group, four mice with APS, and four mice with APS treated with Pam3CSK4. APS mice with established high aPL titers were injected intraperitoneally with Pam3CSK4 at a dose of 1 mg/kg. After 24 h, animals were sacrificed via cervical dislocation and rapid decapitation. Blood samples were collected and processed for PBMC isolation using Histopaque density gradient centrifugation. Monocyte‐enriched fraction was isolated from mouse PBMCs with magnetic bead‐based MojoSort Mouse Monocyte Isolation Kit (BioLegend). Cells were stored in RNAlater at −20°C until mRNA assessment of *TF* and *NLRP3* mRNA expression.

### 2.17 QRT‐PCR

To analyze the mRNA expression of the genes of interest, RNA fraction was isolated with TRIzol reagent (Sigma). Synthesis of cDNA and determination of gene expression levels of *NLRP3*, *TRIF*, *Caspase‐1*, *NF‐kB, PAR2, NOX2*, and *MyD88* in THP‐1, as well as *TF* and *NLRP3* in the mouse monocyte‐enriched fraction, was performed with SOLIScript 1‐step Multiplex Probe Kit (Solis BioDyne), according to manufacturer instructions. *ACTIN* and *GAPDH* served as housekeeping reference genes for THP‐1 cells and the mouse monocyte‐enriched fraction, respectively. PCR primers provided in Table S1. Relative fold changes in gene expression were calculated using the comparative 2^−ΔΔCT^ method.

### 2.18 Assessment of Platelet Aggregations in Tail Blood

To evaluate ex vivo platelet aggregations, blood was collected from the tail vein of mice after the indicated treatments. For morphological examination, peripheral blood smears were prepared immediately as thin blood films on clean glass slides. After air‐drying, the smears were fixed with methanol and stained according to the Pappenheim method [24], and examined using a BOECO BM‐800 light microscope (Germany). Platelet aggregates were assessed under oil immersion at a maximum magnification of 1200×. Platelet aggregation was identified morphologically as the presence of platelet clusters or platelet‐rich aggregates within the blood smear containing at least 20 platelets.

### 2.19 Doppler Ultrasonography Assessment of Vascular Occlusion

Doppler ultrasonography was used to evaluate blood‐flow patency and thrombotic vessel occlusion in mice. Following anesthesia in accordance with the approved institutional animal protocol, mice were positioned on a temperature‐controlled imaging platform, and the anatomical region containing the target vessel was visualized using B‐mode ultrasound. After identification of the vessel, color Doppler imaging was performed to assess intravascular blood flow. Imaging parameters were kept as consistent as possible across animals and experimental groups. For each mouse, Doppler video recordings were acquired from the target vessel.

The presence of red or blue intravascular color signal was considered evidence of detectable blood flow, whereas sustained absence of color Doppler signal in the vessel region was considered compatible with vascular occlusion. Intermittent or weak Doppler signals were classified as residual flow and interpreted as incomplete occlusion or partial flow obstruction. To minimize false classification, the absence of flow was confirmed by repeated visualization. Analyses were performed under blinded conditions.

### 2.20 Statistics

Data analysis was performed with GraphPad Prism v 9.3.1 software (GraphPad Software, USA). Cytometric data were analyzed using FlowJoTM (version 10.1; BD Biosciences). Significance was determined by one‐way ANOVA with Tukey's multiple comparisons test, unless otherwise specified. Statistical parametric analyses were performed following confirmation of the normal distribution of the data. All values are given as mean ± standard error of the mean (SEM), unless otherwise specified. Values of *p* < 0.05 were considered statistically significant.

## 3 Results

### 3.1 TLR‐Ligands Screening for Induction of a Pro‐Coagulatory Shift in aPL‐Stimulated THP‐1 Cells

**FIGURE 1:**
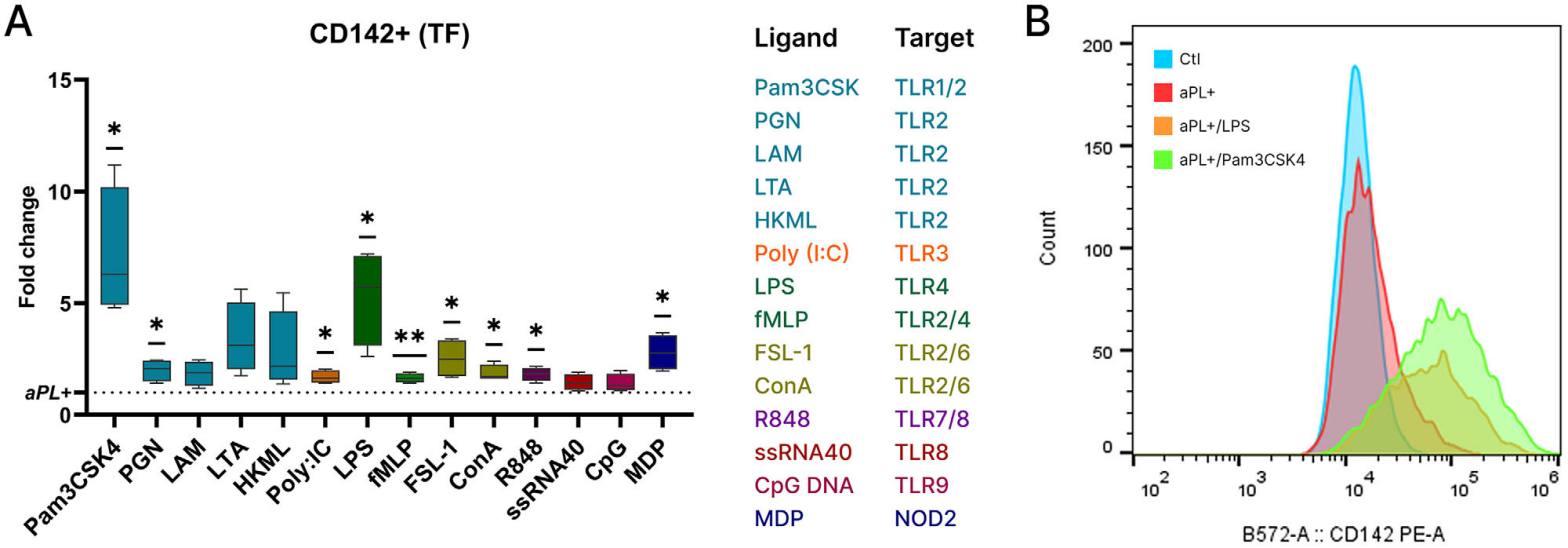
Screening of pathogen‐associated immune stimuli identifies Pam3CSK4 as a strong enhancer of tissue factor (TF, CD142) expression in aPL‐stimulated THP‐1 cells. (A) THP‐1 cells were stimulated with aPL+ IgG in combination with different pathogen‐associated immune stimuli targeting distinct innate immune receptors. Surface TF expression was assessed by flow cytometry using CD142 as a surface marker. Box plots show median and interquartile range, with whiskers indicating minimum and maximum values (*n* = 4). Data are presented as fold change in the percentage of CD142+ cells relative to the aPL+ condition (dotted line). The ligand‐receptor targets used in the screening are shown on the right. Statistical significance was assessed by a one‐sample *t*‐test against the normalized aPL+ baseline. *p < 0.05 vs. aPL+. (B) Representative flow cytometry histogram showing surface CD142 expression on THP‐1 cells stimulated with aPL+ IgG in the presence or absence of LPS and PAM3CSK4.

To assess the pathogenic relevance of different TLR ligands in APS, we employed an in vitro model to screen a range of ligands for their capacity to enhance the pro‐coagulatory effects of anti‐β2GPI antibodies. TF (detected on the cell surface as CD142) was selected as the primary screening readout because it is a central initiator of intrinsic coagulation, a well‐established marker of procoagulant monocyte activation, and a mechanistically relevant indicator of thrombosis‐associated pathways in APS [4, 25, 26]. The presence of aPL antibodies itself promotes a significant increase in the percentage of TF‐positive cells compared with untreated cells, both in vivo and in vitro, as shown by our and numerous other studies [7, 26]. We tested a total of 14 compounds, including an infectious‐origin or synthetic TLR‐ligands and TLR‐inducible ligands for their ability to modulate aPL+ induced TF response. The presence of each of the investigated TLR‐ligands resulted in an additive effect on the aPL+ stimulated rise of TF, albeit to a different extent (Figures 1A,B). In addition to LPS, a well‐known inducer of TF [22], the TLR1/2 ligand Pam3CSK4 was identified as one of the strongest inducers of TF expression. Notably, Pam3CSK4 induced the strongest overall response, reaching a 7.1‐fold increase over the aPL‐only condition, whereas LPS also produced a pronounced effect, with a 5.3‐fold increase. As shown in Figure 1A, LTA (3.4‐fold), HKML (2.8‐fold), FSL‐1 (2.5‐fold), and MDP (2.8‐fold) produced a more moderate enhancement in TF expression. In contrast, PGN (twofold), LAM (1.8‐fold), Poly I:C (1.7‐fold), fMLP (1.6‐fold), ConA (1.8‐fold), R848 (1.8‐fold), ssRNA40 (1.4‐fold), and CpG (1.4‐fold) exerted only minimal or weak effects relative to aPL alone. Therefore, Pam3CSK4 was selected as the ligand of interest for a more detailed investigation into the molecular mechanisms underlying its impact in the context of aPL antibody‐mediated responses.

### 3.2 APL and Pam3CSK4 Synergize to Induce TLR2/TLR4‐Dependent Activation of THP‐1 Cells

**FIGURE 2:**
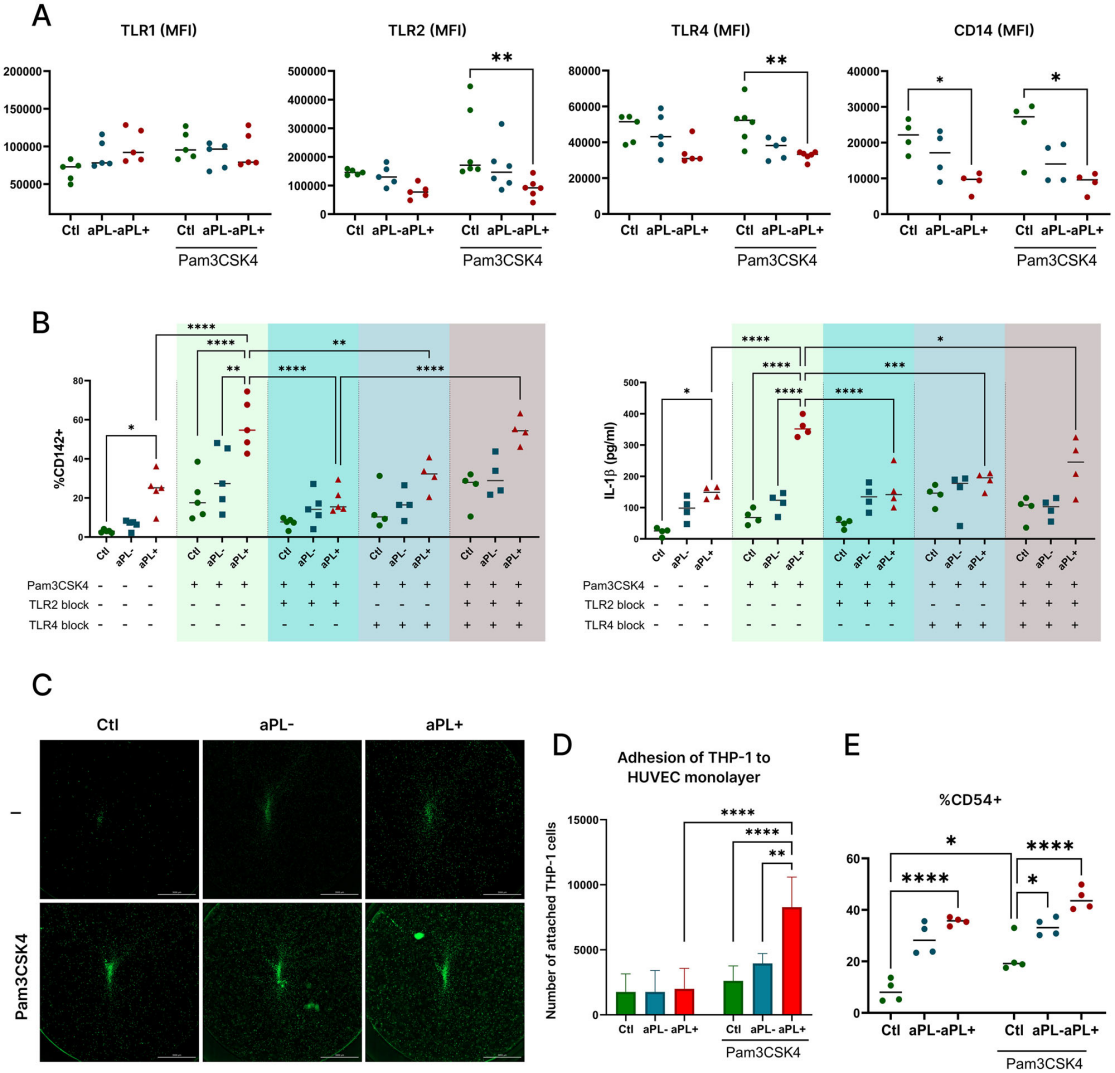
Modulation of THP‐1 cell activation and surface marker expression by aPL+ and Pam3CSK4. (A) Surface expression of TLR1, TLR2, TLR4, and CD14 on THP‐1, measured by flow cytometry and shown as mean fluorescent intensity (MFI). (B) Effects of TLR blockade on tissue factor (TF, CD142) expression and IL‐1β secretion in THP‐1 cells stimulated with Pam3CSK4 (200 ng/mL) and/or aPL−/aPL+. THP‐1 cells were pretreated with TLR2 signaling inhibitor (TL2‐C29, 100 µM), TLR4 signaling inhibitor (CLI‐095, 1 µM), alone or in combination, followed by stimulation with Pam3CSK4, aPL−, aPL+, or their combination for 4 h. Surface expression of TF (CD142) was measured by flow cytometry, and IL‐1β levels were quantified in culture supernatants by ELISA. (C) Representative merged images of carboxyfluorescein succinimidyl ester (CFSE) labeled THP‐1 cells attached to HUVEC cell monolayer under dynamic conditions (*n* = 5). (D) A quantitative assessment (*n* = 5) of the number of fluorescent THP‐1 cells adhering to HUVECs performed using a fluorescence microscope. (E) Percentage of CD54+ cells detected on THP‐1 cells.

To define the receptor context associated with the response to aPL and Pam3CSK4, we assessed both the surface expression and the functional involvement of candidate TLRs. TLR1 and TLR2 were examined because Pam3CSK4 signals through the TLR1/TLR2 heterodimer, whereas TLR4 was included in view of previous evidence implicating TLR4 in aPL‐induced monocyte activation to TLR4‐dependent signaling. Accordingly, the surface expression of TLRs was evaluated by flow cytometry (Figure 2A), and receptor‐blocking experiments were performed to assess their contribution to the enhanced procoagulant response and IL‐1β release induced by co‐stimulation (Figures 2B).

Analysis of TLRs expression following stimulation revealed differential regulation of the TLR1/TLR2 heterodimer (Figure 2A). While TLR1 expression was unchanged in response to aPL+ and Pam3CSK4, its dimerization partner, TLR2, was significantly downregulated in the presence of both stimuli. The observed imbalance in heterodimer expression may be attributed to the differential turnover and dynamics of TLR1 and TLR2, as demonstrated in various inflammatory contexts [27]. A similar downregulation pattern was observed for TLR4 (Figure 2A), suggesting potential receptor internalization or shedding following activation. Given the affinity of the anti‐β2GPI/β2GPI complex for TLR4 and the described shedding of TLR4 in its presence, we also analyzed the surface expression of CD14, which acts as a regulator of TLR4 internalization. This internalization process is essential for initiating the TRIF‐dependent signaling pathway downstream of TLR4 activation. Once in the endosomes, TLR4 associates with a secondary set of adaptor proteins, namely the TRAM and TRIF, leading to type I IFN production and contributing to the late activation of NF‐κB [28]. In our study, stimulation with aPL+ led to a significant decrease in CD14 surface expression in comparison to the control group (Figure 2A). Notably, co‐stimulation with Pam3CSK4 did not exert any additive effect on aPL‐induced CD14 downregulation, mirroring the pattern observed for TLR4 expression.

Next, we aimed to investigate the functional relevance of TLR signaling by selectively blocking TLR pathways. This approach allowed us to assess the contribution of individual receptors to the pro‐inflammatory activation of THP‐1 cells. TLR inhibition was performed using synthetic signaling inhibitors (TL2‐C29, which targets TLR2/1 and TLR2/6 pathways; and CLI‐095, a TLR4 signaling inhibitor). Overall, the signaling inhibition strategy was effective in suppressing dual Pam3CSK4− and aPL+ IgG‐mediated activation of THP‐1 cells. Particularly, blocking TLR2 signaling demonstrated significantly reduced both the percentage of CD142+ cells and the levels of IL‐1β induced by combined Pam3CSK4 and aPL+ stimulation (Figure 2B). Similarly, inhibition of TLR4 signaling with CLI‐095 led to a significant reduction in both CD142‐expressing cells and IL‐1β production in Pam3CSK4 and aPL+ stimulated cells. Simultaneous blockade of TLR2 and TLR4 markedly decreased only the IL‐1β secretion without significantly affecting CD142 expression in double‐stimulated cells. Moreover, the percentage of CD142+ cells was higher following combined TLR2 and TLR4 inhibition compared with TLR2 blockade alone. Thus, combined TLR2/TLR4 inhibition showed a clear inhibitory effect on inflammasome‐related cytokine release, but no additive suppressive effect on the percentage of CD142+ cells. This suggests that IL‐1β secretion and CD142 expression are not regulated identically. While IL‐1β production appears to depend strongly on convergent TLR2/TLR4 signaling, CD142 positivity may be maintained through partially redundant or compensatory pathways once the procoagulant phenotype has been initiated.

To confirm the activation state of cells exposed to dual stimulation, we evaluated their adhesive properties. A functional adhesion assay demonstrated the heightened ability of double‐stimulated with aPL+ and Pam3CSK4 THP‐1 cells to adhere to HUVEC cell monolayer, which reflects increased activation and integrin‐mediated adhesion (Figure 2C,D). The upregulation of CD54 (Figure 2E) supported the notion that THP‐1 cells become activated in response to combined stimulation. Representative histograms are shown in Figure S1.

### 3.3 APL+ and Pam3CSK4 Activate THP‐1 Cells via the NLRP3 Inflammasome

Since IL‐1β processing is a hallmark of inflammasome activation [29], we next examined the NLRP3 pathway as a potential mechanism mediating this response.

**FIGURE 3:**
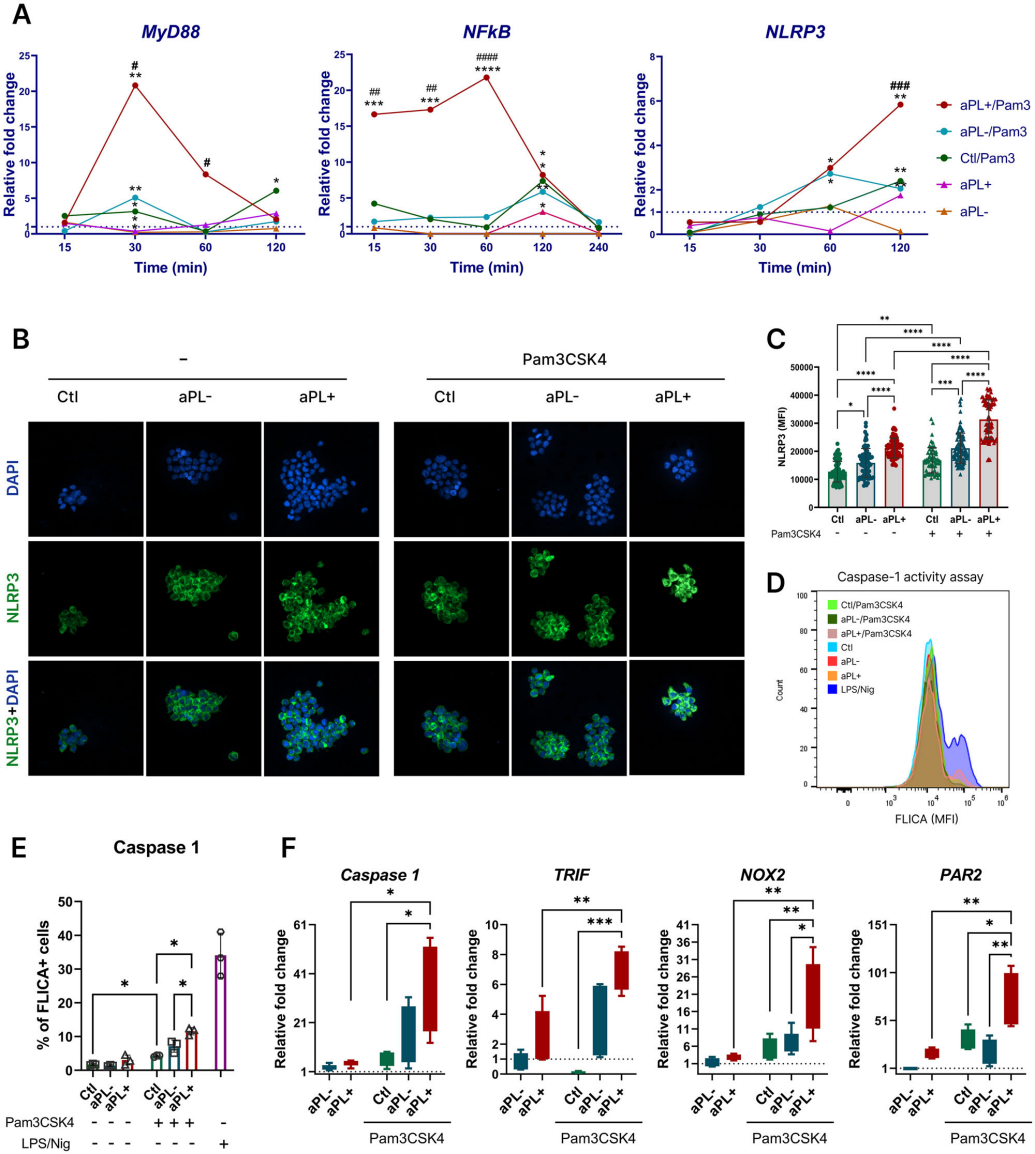
Pam3CSK4 enhances inflammasome‐related signaling in aPL‐stimulated THP‐1 cells. (A) Time‐dependent changes in *MyD88*, *NFkB*, and *NLRP3* mRNA response in THP‐1 cells following stimulation (*n* = 5). The dotted line indicates the expression level in unstimulated control cells. Statistical significance versus the normalized unstimulated control was assessed by a one‐sample *t*‐test. (B) Representative immunofluorescence images of THP‐1 cells showing nuclei (blue) and NLRP3 (green) expression (*n* = 3). (C) Quantification of NLRP3 mean fluorescence intensity (MFI) calculated using microscopy image‐analysis software from five randomly selected fields per condition in each independent experiment (*n* = 3). Data are shown as mean ± SD. Statistical significance was assessed using the Kruskal–Wallis test followed by Dunn's multiple‐comparison test. (D) Representative flow cytometry histogram showing caspase‐1 activity in the studied groups. (E) Caspase‐1 activity measured with FLICA assay and expressed as the % of FLICA+ THP‐1 cells. (F) mRNA levels of *Caspase‐1*, *NOX2*, and *PAR2* following 1 h treatment, and *TRIF* after 4 h stimulation. Box plots show median and interquartile range, with whiskers indicating minimum and maximum values (*n* = 5). The dotted line indicates the expression level in unstimulated control cells. Expression of β‐actin was used as a reference to calculate the relative expression of target genes with the 2^−ΔΔCt^ method. *p < 0.05, **p < 0.01, ***p < 0.001*. #p < 0.05, ###p <* 0.001 ‐ difference between aPL‐/Pam3CSK4 and aPL+/Pam3CSK4 groups.

Time‐course analysis of gene expression revealed a sequential activation of inflammatory signaling pathways. Gene expression monitoring of single and double‐stimulated cells revealed the sequential activation of signaling pathways. Exposure of THP‐1 cells separately by aPL+ or Pam3CSK4 induced an early upregulation of *MyD88* and *NF‐κB* within the first 120 min (Figure 3A). However, when cells were exposed to aPL+ together with Pam3CSK4, their combined presence led to a markedly stronger and earlier activation of *NF‐κB*, observable as early as 60 min poststimulation, ultimately resulting in a significant increase in *NLRP3* expression (Figure 3A). Consistent with these transcriptional findings, immunofluorescence analysis performed after 4 h of stimulation demonstrated increased NLRP3 staining of THP‐1 cells exposed to the combined treatment (Figure 3B,C). As NLRP3 serves as a crucial component of the inflammasome complex, its upregulation suggested enhanced priming of the NLRP3 pathway. Upon priming, NLRP3 oligomerizes with the adaptor protein ASC, promoting the activation of caspase‐1, which subsequently cleaves pro‐IL‐1β into its mature, secreted form. In line with this mechanism, co‐stimulation with aPL and Pam3CSK4 was associated with increased caspase‐1 activity (Figures 3D, E) and upregulation of *Caspase‐1* mRNA expression (Figure 3F).

Mechanisms of aPL‐induced inflammasome activation are not limited to TLR4‐mediated signaling. Previous studies have shown that aPL can enter the lysosomal pathway in monocytes and dendritic cells, leading to activation of endosomal NADPH oxidase (NOX2) [30]. With the employment of human‐derived, cofactor‐independent aPL antibodies, it was demonstrated that NLRP3 activation and IL‐1β production are largely dependent on NOX2 activity and can occur independently of TLR2 and TLR4 signaling [31]. Besides, a link between aPL‐induced endosomal NOX2 activation and expression of TF was proposed [30]. We also observed a threefold increase in *NOX2* expression in THP‐1 cells stimulated with aPL alone, which was further enhanced up to 16‐fold upon co‐stimulation with Pam3CSK4. Upregulated *NOX2* expression further supports the engagement of multiple signaling pathways in response to dual stimulation (Figure 3F).

mRNA level of *PAR2*, receptor known to play an important role in aPL‐induced TF expression [32] and fetal injury [33], was upregulated in the cells exposed to the combined presence of aPL+ or Pam3CSK4 compared with cells treated with aPL+ alone (Figure 3F). Similarly, the expression of *TRIF*, an adaptor molecule incorporated in TLR4‐TRIF and PAR2‐TRIF alternative signaling axes [34], reached the highest level following 4 h combined stimulation with aPL+ IgG and Pam3CSK4 (Figure 3F).

### 3.4 Dual Stimulation With aPL and Pam3CSK4 Enhances Inflammasome‐Associated Effector Responses

**FIGURE 4:**
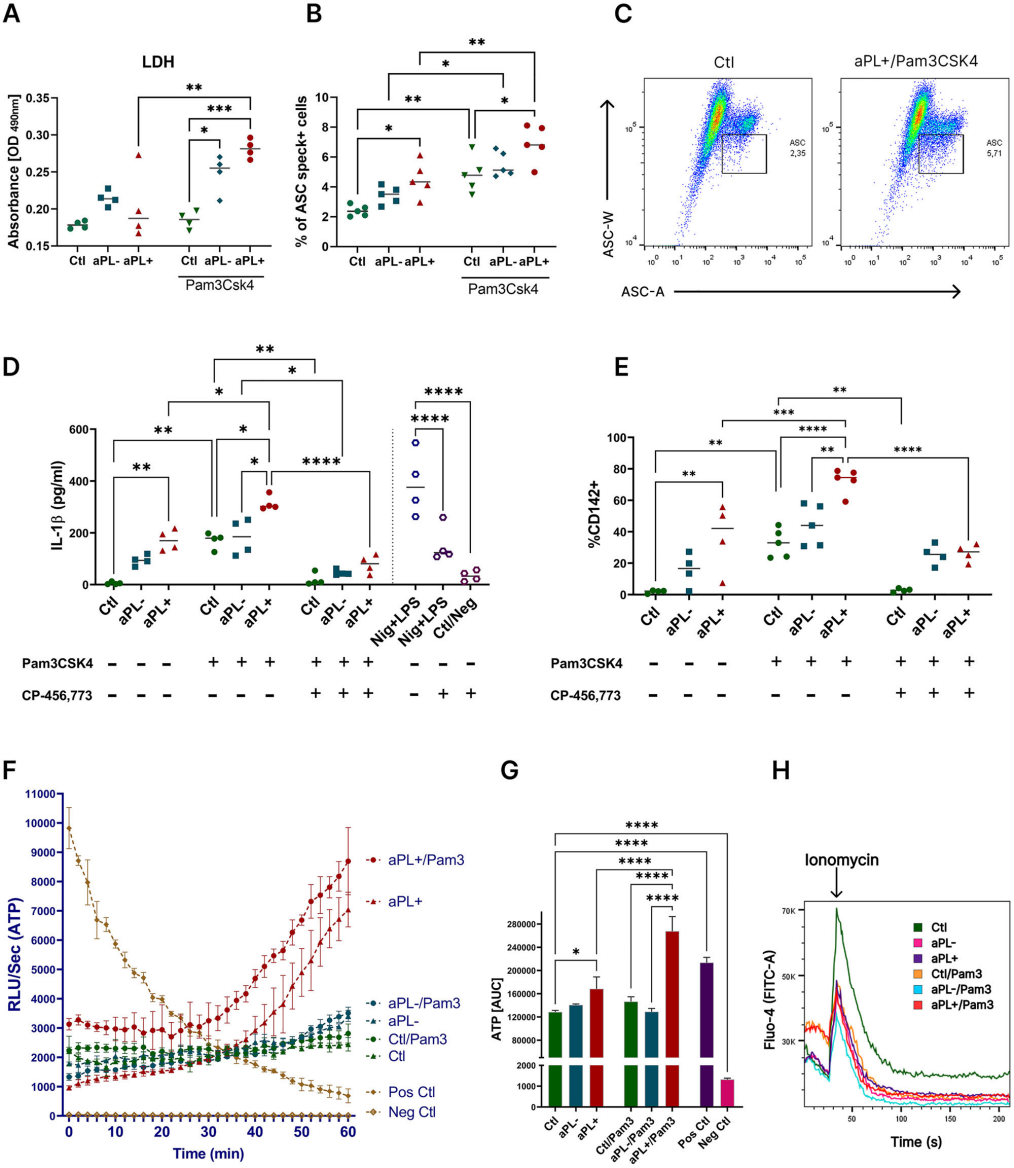
Functional evidence of aPL/Pam3CSK4‐induced NLRP3 inflammasome activation and TF upregulation in THP‐1 cells. (A) Lactate dehydrogenase (LDH) levels measured in the culture supernatants as an indicator of cytotoxicity. (B) Quantification of ASC speck‐positive cells by flow cytometry. (C) Representative dot plot showing ASC speck formation in THP‐1 cells. (D) Concentrations of IL‐1β in the supernatants of the cells stimulated with aPL+, aPL−, and Pam3CSK4, measured by ELISA. Nigericin (Nig) was used as a positive control for NLRP3 activation, and CP‐456,773 as a selective inhibitor of NLRP3. (E) Percentage of TF/CD142+ THP‐1 cells measured by flow cytometry. (F) Kinetic luminescent quantification of extracellular adenosine triphosphate (ATP) levels released by THP‐1 cells. Luminescence intensity reflects ATP‐dependent luciferase activity. ATP at a final concentration of 0.1 µM was used as a positive control, and cell‐free RPMI as a negative (*n* = 3). (G) Area under curve (AUC) analysis of ATP‐release kinetics shown in panel F, providing cumulative quantification of extracellular ATP release over the measurement period (*n* = 3). Data are shown as mean ± SD. (H) Ca^2^^+^ add‐back assay. Stimulated THP‐1 were loaded with Fluo‐4 AM for 30 min in the absence of extracellular Ca^2^^+^, and then 1 mM Ca^2^^+^ was added for 30 min before measurement. The arrow indicates the time point at which Ionomycin (1 µg/mL) was ad*ded (n* = 3). *p < 0.05, **p < 0.01, ***p < 0.001, ****p < 0.0001.

Following inflammasome activation, two key downstream mechanisms that take place are pyroptosis and cytokine secretion. Pyroptosis is a programmed inflammatory form of cell death triggered by inflammasome‐dependent caspase‐1 activation [35]. To trace the impact of strong NLRP3 activation by aPL+ and Pam3CSK4, we measured the LDH release, an indicator of cell lysis during pyroptosis. Our results indicated that LDH release by cells stimulated with both the aPL+ and Pam3CSK4 is significantly higher than that by cells separately stimulated by aPL+ or Pam3CSK4 (Figure 4A). The evidence for pyroptosis was further supported by the detection of ASC specks, a characteristic indicator of inflammasome assembly. Although the differences in ASC speck formation were within a few percentage points (Figure 4B,C), they were statistically significant and correlated with caspase‐1 and NLRP3 expression levels, as well as IL‐1β release. Measurement of IL‐1β in cell culture supernatants revealed a positive correlation between cell surface CD142 expression and produced IL‐1β (Figure 4D,E). We detected a slight increase in IL‐1β production upon aPL+ exposure. However, comparison of IL‐1β levels revealed that cells co‐stimulated with aPL+ and Pam3CSK4 released the highest amounts of IL‐1β, reaching nearly three times the level induced by aPL+ alone and four times that induced by Pam3CSK4 alone (Figure 4D).

Signals delivered by TLR ligands are essential for licensing NLRP3 inflammasome activation through NF‐κB‐mediated upregulation of NLRP3 expression. Both the aPL+ and Pam3CSK4 can serve as initial priming signals, while a secondary stimulus is required to fully activate the NLRP3 inflammasome. In the current study, we observed a steady time‐dependent increase in ATP release in THP‐1 cells stimulated with aPL+ (Figure 4F,G). The presence of Pam3CSK4 further increased the ATP release initiated by aPL+, suggesting the involvement of an autocrine signaling feedback loop that drives exaggerated inflammatory responses.

Another important factor for the assembly of the NLRP3 inflammasome and the release of IL‐1β is the second messenger, intracellular calcium ion (iCa^2^^+^). We measured both baseline (spontaneous) and ionomycin‐induced changes in iCa^2^^+^ levels. Spontaneous iCa^2^^+^ levels were the highest in the cells treated with Pam3CSK4 alone and Pam3CSK4 with aPL+ IgGs. The response to ionomycin was biphasic, characterized by an initial rapid rise in iCa^2^^+^ levels, reaching a peak followed by a decay phase, although the kinetics of iCa^2^^+^ flux were largely similar across all experimental groups. In unstimulated control THP‐1 cells, Ca^2^^+^ levels returned to baseline during the decay phase. In contrast, cells prestimulated with IgG and Pam3CSK4 exhibited a more pronounced decline, with Ca^2^^+^ levels falling below baseline (Figure 4H). Importantly, Pam3CSK4 and aPL+ stimulation exhibited the highest NLRP3 inflammasome activation and IL‐1β release throughout our study, indicating that elevated basal calcium levels, rather than the extent of ionomycin‐induced calcium influx, may play a more critical role in initiating inflammasome assembly.

### 3.5 Pam3CSK4 Administration Enhances Prothrombotic Responses in APS Model Mice

**FIGURE 5:**
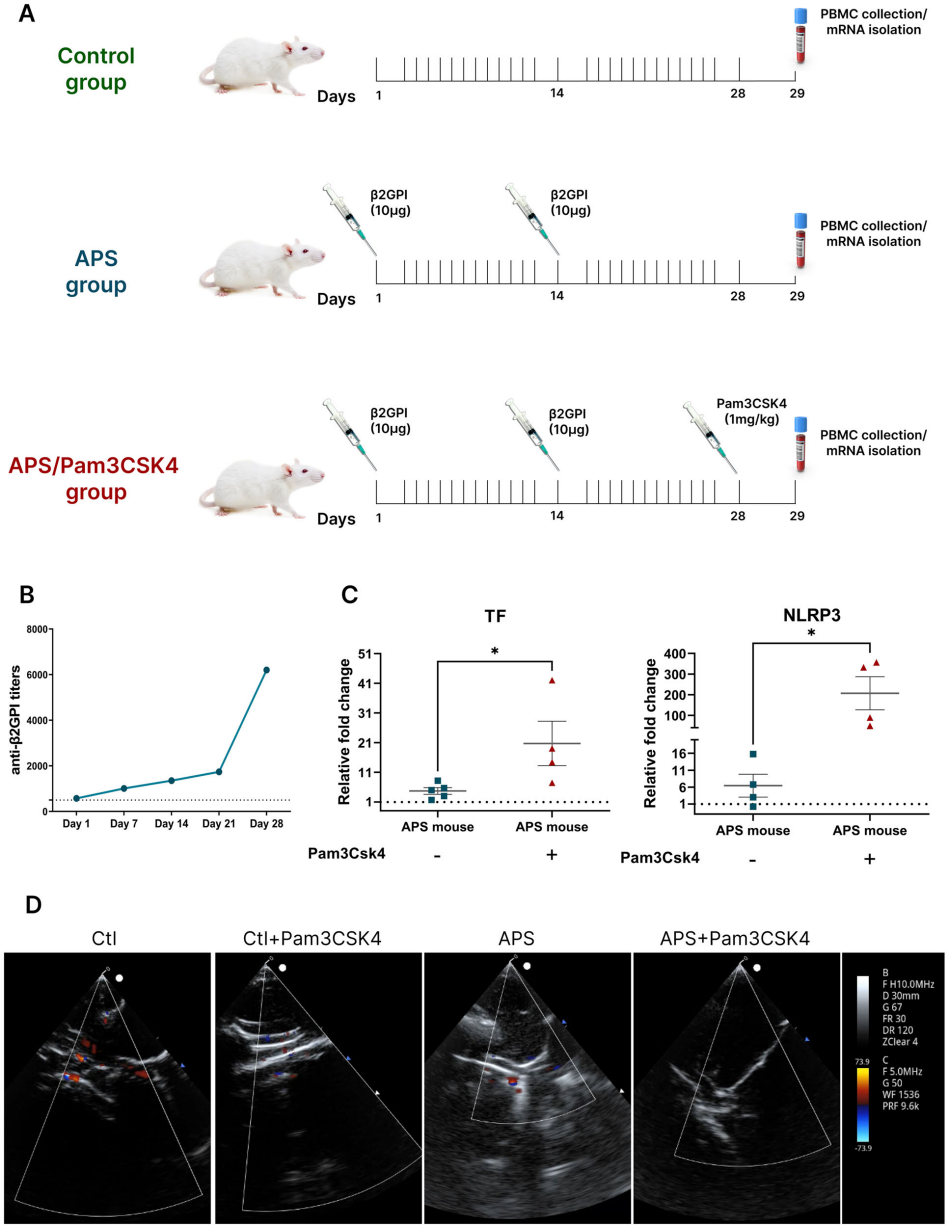
Pam3CSK4 exacerbates thromboinflammatory responses and vascular alterations in the APS mouse model. (A) Schematic representation of the APS mouse model development and timeline of Pam3CSK4 treatment. (B) Quantification of anti‐β2GPI antibody titers in the peripheral blood of APS mice. (C) Relative mRNA expression levels of NLRP3 and tissue factor (TF) in monocyte‐enriched fraction isolated from APS mice compared with healthy controls. *GAPDH* served as a housekeeping reference gene. Relative fold changes in gene expression were calculated using the comparative 2^−ΔΔCT^ method. Statistical significance was assessed using an unpaired two‐tailed *t*‐test. (D) Representative Doppler ultrasound images showing intrahepatic vascular perfusion patterns in healthy, Pam3CSK4‐treated, APS, and APS/Pam3CSK4‐treated mice (*n* = 2 in each group).

To validate whether the aPL/Pam3CSK4 interaction observed in vitro translates into a physiologically relevant in vivo response, we employed an APS mouse model, as recently described by Martirosyan et al. [23]. The experimental design and Pam3CSK4 treatment schedule are shown in Figure 5A. Following confirmation of APS induction, evidenced by elevated anti‐β2GPI antibody titers (Figure 5B), the mice were injected with Pam3CSK4 according to the experimental scheme (Figure 5A). Subsequent analyses demonstrated a prothrombotic shift, evidenced by upregulated *TF* expression in APS mice monocyte‐enriched fraction in response to Pam3CSK4 (Figure 5C). Furthermore, increased mRNA levels of *NLRP3* suggested enhanced inflammasome pathway priming following Pam3CSK4 stimulation in vivo in aPL+ animals, complementing the functional inflammasome‐related findings obtained in vitro (Figure 5C).

To further assess thromboinflammatory consequences in vivo, platelet aggregation was evaluated in blood smears. Healthy mice and Pam3CSK4‐treated control mice showed low numbers of platelet aggregates, with 16 and 20 aggregates, respectively. APS mice showed increased platelet aggregation, with 48 aggregates counted. The highest number of aggregates was observed in APS mice treated with Pam3CSK4, reaching 73 aggregates and significantly exceeding all other studied groups (Figure S2A,B). These data indicate that Pam3CSK4 enhances the proaggregatory platelet response in the APS setting.

Liver color Doppler examination further revealed heterogeneous intrahepatic vascular perfusion among the analyzed groups. In healthy mice, color Doppler imaging showed clearly detectable intrahepatic vascular flow. Several vessel‐like structures demonstrated visible red and blue Doppler signals, indicating preserved blood flow within the examined liver region (Figure 5D; Videos S1–S4). No clear evidence of large‐vessel obstruction was observed. In APS mice, the color Doppler signal was detectable but appeared patchy and discontinuous. Some vascular branches showed red and blue flow signals; however, the signal was less extensive than that observed in healthy mice. Larger vessel structures remained visible and did not show consistent evidence of complete occlusion. These findings suggest partial preservation of intrahepatic flow, with reduced Doppler signal mainly at the level of small intrahepatic vessels. In mice injected with Pam3CSK4, the color Doppler signal was present but appeared less intense and more scattered compared with healthy mice. Small intrahepatic vessels showed detectable but relatively limited color filling. Larger vessel structures remained visible, and there was no stable evidence of complete large‐vessel flow loss. Overall, this pattern suggests preserved but weaker detectable intrahepatic perfusion. And finally, APS mice treated with Pam3CSK4 showed the most pronounced reduction in liver Doppler signal. Vessel‐like structures could be identified on B‐mode imaging, but color filling was minimal or nearly absent in many regions. This pattern may indicate markedly reduced detectable intrahepatic perfusion, particularly at the level of small vessels. Thus, across the studied groups, the most apparent Doppler differences were observed in small intrahepatic vessels. Doppler signals ranged from clearly preserved in healthy mice to markedly reduced or nearly absent in APS mice treated with Pam3CSK4, whereas larger intrahepatic vessels did not show consistent evidence of complete occlusion.

Together, the increased *TF* expression, enhanced NLRP3 pathway priming, elevated platelet aggregation, and reduced small‐vessel Doppler signal support the conclusion that Pam3CSK4 amplifies thromboinflammatory alterations in APS mice in vivo. These findings complement the mechanistic in vitro data and support the pathophysiological relevance of the aPL/Pam3CSK4 interaction.

## 4 Discussion

Antiphospholipid antibodies have a well‐defined association with a high risk of recurrent thromboembolic events [36], and infection itself is an independent potential risk factor for thrombosis [37]. In this context, we identified Pam3CSK4, alongside LPS, as a potent inducer of TF expression, supporting the idea that infection‐related innate immune ligands may amplify the responsiveness of aPL‐primed cells. This is biologically plausible, as Pam3CSK4 is a synthetic triacylated lipopeptide that mimics bacterial lipoproteins and is considered the most well‐known TLR1/2 agonist [38, 39].

In the present study, for the first time, we have demonstrated the potential of Pam3CSK4 to synergize with anti‐β2GPI antibodies to elicit a robust proinflammatory and prothrombotic response from monocyte‐like cells. Our observation clearly pointed out that the presence of a second stimulus, specifically engaging TLR1/TLR2 receptors, has the potential to dramatically multiply the IL‐1β response of aPL‐stimulated cells. As appeared, signaling initiated by Pam3CSK4 in aPL‐primed cells was mediated through the NF‐κB pathway and the NLRP3 inflammasome. In the context of Pam3CSK4 signaling, the engagement of TLR1/2 activated the NLRP3 inflammasome, which in turn amplified the inflammatory response, promoting the maturation and release of IL‐1β. The NLRP3 inflammasome is a multi‐protein complex that includes the sensor molecule NLRP3, the adaptor protein ASC, and the protease caspase‐1 [29, 40], all of which have been implicated in the mechanisms observed in our study. Activation of NLRP3 is considered a two‐step process: an initial priming step required for increased expression of components and substrates of the NLRP3 inflammasome and an activation stimulus step that elicits inflammasome formation. The second agent is triggered by a range of pathogen‐ and danger‐associated molecules, with extracellular ATP being one of the key stimuli [29, 40]. Our findings indicate that aPL induced a significant release of ATP from the cells, suggesting its potential role in the activation of the inflammasome. Although the exact mechanism behind ATP release remains unclear. First, aPL might trigger the activation of ion channels, such as P2X or P2Y purinergic receptors. aPL/β2GPI binding on the cell membrane may induce lipid raft clustering, potentially influencing purinergic receptor localization or function, thereby facilitating ATP release [17, 41]. In addition, given the affinity of anti‐β2GPI for TLR4, TLR4/P2×7 crosstalk may also play a role in induced ATP release [42].

The procoagulant arm of the response is likewise biologically credible. Inflammation is known to disrupt hemostatic equilibrium and shift it toward a pro‐thrombotic state, posing a significant risk for patients with coagulopathies [43, 44]. TLR2/Pam3CSK4 signaling has been linked to TF induction in monocytic/macrophage systems [45, 46], and APS‐related studies have independently shown that aPL can drive TF expression through TLR2‐ or TLR4‐linked pathways [14, 25, 47, 48]. Particularly relevant to APS, disruption of the TFPI‐regulated cell‐surface TF complex has been proposed as a mechanism through which aPL facilitates rapid prothrombotic signaling in monocytes [49]. Against this background, the increase in TF observed after combined aPL and Pam3CSK4 stimulation is best interpreted as amplification of an already permissive procoagulant state, rather than as evidence that Pam3CSK4 alone universally induces thrombosis. Emerging evidence suggests that excessive pyroptosis promotes the release and coagulation activity of TF [50]. Specifically, plasma membrane pore formation during programmed pyroptosis not only facilitates the release of pro‐inflammatory intracellular contents, including TF, but also promotes TF decryption [51], a posttranslational modification that dramatically amplifies its procoagulant activity [52]. In mouse models, pyroptotic monocytes and macrophages contributed to systemic coagulation and venous thrombosis, while tissue factor deficiency was protective [53]. These observations make pyroptosis a plausible bridge between inflammatory signaling and TF‐dependent coagulation in our system. However, this link should be considered a mechanistic hypothesis supported by adjacent thrombosis literature rather than a fully established APS‐specific mechanism.

Our findings fit well within the broader “second‐hit” concept in APS [7, 54]. In vivo and translational APS studies already support the idea that innate immune triggers can determine whether aPL positivity remains subclinical or evolves into overt thromboinflammation [55, 56]. LPS/TLR4‐dependent models are the clearest precedent [21, 22], as aPL‐mediated thrombosis and cellular activation are reduced in TLR4‐deficient mice, and LPS has been proposed to act as a second hit by enhancing β2GPI expression and promoting aPL‐mediated endothelial activation [13, 21, 22]. Clinically, low‐grade endotoxemia has also been associated with oxidative stress and recurrent thrombosis in patients with primary APS [57]. Our data extend this concept to the TLR1/TLR2 axis, consistent with evidence that TLR2/CD14 can mediate aPL‐induced activation of human monocytes and endothelial cells [47]. Moreover, previous studies have linked aPL to inflammasome‐related responses, including NLRP3 activation and synergistic induction of IL‐1β/caspase‐1 responses in the presence of selected TLR ligands [31, 58]. Therefore, Pam3CSK4 may represent a TLR1/TLR2‐dependent inflammatory second hit capable of amplifying aPL‐induced proinflammatory and procoagulant activation. However, because Pam3CSK4 effects are context‐dependent and direct clinical data linking TLR1/TLR2‐activating infections to more severe APS manifestations are lacking, this interpretation should be considered mechanistic rather than epidemiologically proven.

These findings also highlight potential therapeutic targets. Strategies aimed at limiting TLR signaling, particularly through anti‐TLR1/2 or anti‐TLR4 pathways, may offer promising avenues to mitigate pathological monocyte responsiveness during infectious episodes. Although such approaches are not applicable to routine APS management, they may be relevant in selected severe settings, such as infection‐complicated APS or catastrophic antiphospholipid syndrome [59, 60]. Supporting this concept, anti‐TLR2 or anti‐TLR4 monoclonal antibodies conferred protection in murine models of severe polymicrobial sepsis [61], potentially by suppressing inflammatory and procoagulant pathways analogous to those observed in our study.

A major limitation of this study is the use of THP‐1 cells for in vitro mechanistic experiments. Although this model provides a reproducible platform for studying inflammatory signaling, it does not fully reflect the heterogeneity and complexity of primary human monocytes. While key findings were supported in vivo, further validation in primary human cells is warranted.

In summary, our findings support the “second hit” paradigm of APS, highlighting that microbial components sensed by TLR1/2 may contribute to disease exacerbation in aPL‐positive individuals. For aPL‐positive individuals, the pathological implications extend beyond the modest secretion of IL‐1β. The chronic presence of aPL may prime immune cells, enhancing their responsiveness to subsequent TLR stimulation and thereby promoting exaggerated inflammatory and pro‐thrombotic responses, potentially precipitating clinical manifestations and increasing mortality in cases of infection‐associated APS. The current study reinforces the notion that infectious diseases might pose a significant risk to aPL‐positive individuals. Our findings uncover a previously unrecognized molecular mechanism whereby TLR1/2 ligands, and potentially infection‐related stimuli, drive amplified inflammatory responses in aPL‐primed cells, providing new insight into the pathogenesis of APS. The findings underscore the complexity of monocyte activation pathways in APS and suggest combinatorial engagement of pattern recognition receptors, which may underlie the robust inflammatory and procoagulant phenotype observed in infection‐complicated cases of the disease.

## Author Contributions

**Anush Martirosyan**: investigation; methodology; formal analysis; conceptualization, visualization; Writing – original draft preparation. **Eva Kriegova**: funding acquisition; Writing – review and editing. **Jana Ulehlova**: formal analysis; visualization. Zaven Karalyan: methodology; visualization. **Tomas Papajik**: funding acquisition; Writing – review and editing. **Gayane Manukyan**: Investigation; methodology; conceptualization; funding acquisition; Writing – original draft Preparation; Writing – review and editing. All authors read the final version of the manuscript.

## Funding

This work was supported by the State Committee of Science MES RA, in the frame of the research project no. SCS 21AG‐1F072, and in part by the Ministry of Health of the Czech Republic (FNOl, 00098892) and the European Regional Development Fund‐Project (No.CZ.02.01.01/00/23_021/0009224) co‐funded by the European Union.

## Conflicts of Interest

The authors declare no conflicts of interest.

## Supporting information




**Supporting File**: eji70245‐sup‐0001‐SuppMat.zip.

## Data Availability

The data that support the findings of this study are available in the figures and Supporting Information of this article.
