# Supplementary material for: Pam3CSK4 As a Second Hit in NLRP3‐Dependent Activation of Monocytes Primed With Antiphospholipid Antibodies
Source: Eur J Immunol. 2026 Jul 22;56(7):e70245. doi: 10.1002/eji.70245 (PMC13390583; doi:10.1002/eji.70245)
Supplement: Supplementary file 1 — Supporting File: eji70245‐sup‐0001‐SuppMat.zip. [file EJI-56-e70245-s001.zip › eji70245-sup-0001-SuppMat.docx]

**Pam3CSK4 as a second hit in NLRP3-dependent activation of monocytes primed with antiphospholipid antibodies**

**Table S1.** Primer sequences for human and mouse genes.

| **Human Genes** |  |  |
| --- | --- | --- |
| ***Actin*** | **forward** | CGCGAGAGAAGATGACCCAGATC |
|  | **reverse** | GCCAGAGGCGTACAGGGATA |
| ***NLRP3*** | **forward** | AGAGACCTTTATGAGAAAGCAA |
|  | **reverse** | GCTGTCTTCCTGGCATATCACA |
| ***NFkB*** | **forward** | CTGTCCTTTCTCATCCCATCTT |
|  | **reverse** | ACACCTCAATGTCCTCTTTCTG |
| ***TRIF*** | **forward** | ACCTCCTGCATGCCATGGTTCT |
|  | **reverse** | TCAGCCAGCAGGTGGTACAA |
| ***MyD88*** | **forward** | GGCTGCTCTCAACATGCGA |
|  | **reverse** | CTGTGTCCGCACGTTCAAGA |
| ***PAR2*** | **forward** | AACCAAGCTTTCTCGGTGCGTCCAGT |
|  | **reverse** | GCTCTAGACTGCAATTCCCATCTGAGG |
| ***Caspase1*** | **forward** | AAAATCTCACTGCTTCGGACATG |
|  | **reverse** | GGAACGTGCTGTCAGAGGTCTT |
| ***NOX2*** | **forward** | GGCTTCCTCAGCTACAACATCT |
|  | **reverse** | GTGCACAGCAAAGTGATTGG |
| **Mouse Genes** |  |  |
| ***GAPDH*** | **forward** | TGCGACTTCAACAGCAACTC |
|  | **reverse** | CTTGCTCAGTGTCCTTGCTG |
| ***NLRP3*** | **forward** | TCCTGGTGACTTTGTATATGCGT |
|  | **reverse** | TTCTCGGGCGGGTAATCTTC |
| ***TF*** | **forward** | ATGAGGAGCTGTGTTAAAGGGTCGCAGAA |
|  | **reverse** | TGCAGTAAATGCACGTGTCTGCCAT |

**
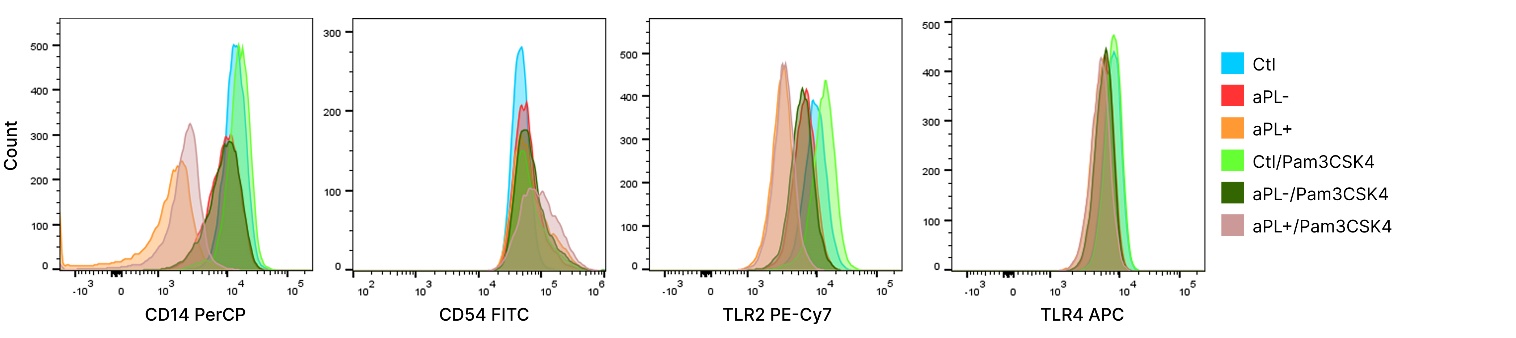
**

**Suppl. Figure 1.** Representative flow cytometry histograms showing surface expression of CD14, CD54, TLR2, and TLR4 in THP-1 cells cultured in the presence of aPL and Pam3CSK4.

**
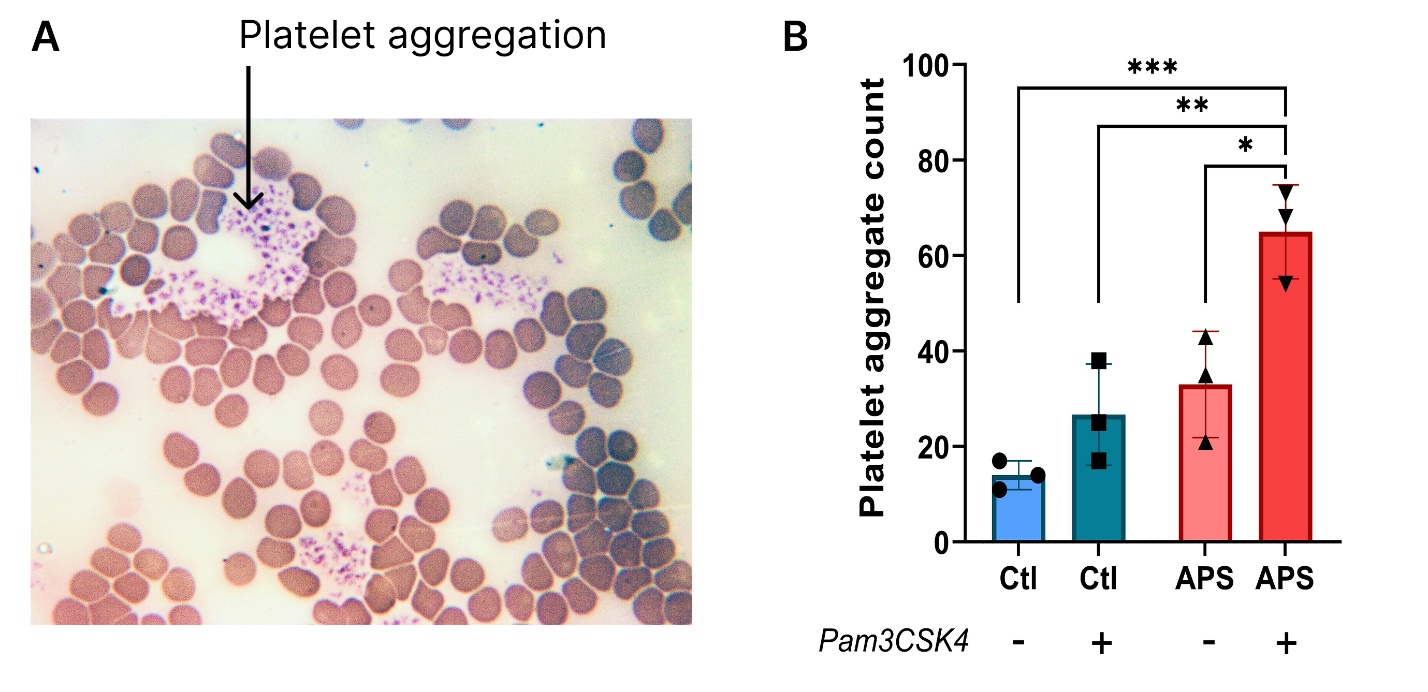
**

**Suppl. Figure2. Assessment of platelet aggregations in tail blood of experimental mice. A)** Representative light microscopy image showing platelet aggregates in a blood smear. Blood smears were stained using the Pappenheim method. Platelet aggregates were assessed under oil immersion at a maximum magnification of 1200×; **B)** Quantification of platelet aggregates in tail blood smears from experimental mice (n=3). Data are shown as mean ± SD. Statistical analysis was performed using one-way ANOVA with Tukey’s multiple comparisons test. *p< 0.05, **p< 0.01, ***p< 0.001.
